# The utility of texture analysis based on quantitative synthetic magnetic resonance imaging in nasopharyngeal carcinoma: a preliminary study

**DOI:** 10.1186/s12880-023-00968-w

**Published:** 2023-01-25

**Authors:** Fan Yang, Yujie Li, Xiaolu Li, Xiaoduo Yu, Yanfeng Zhao, Lin Li, Lizhi Xie, Meng Lin

**Affiliations:** 1 Department of Diagnostic Radiology, National Cancer Center/National Clinical Research Center for Cancer/Cancer Hospital, Chinese Academy of Medical Sciences and Peking Union Medical College, Beijing, 100021 China; 2 MR Research China, GE Healthcare, Beijing, China

**Keywords:** Nasopharyngeal carcinoma, Magnetic resonance imaging, Differential diagnosis

## Abstract

**Background:**

Magnetic resonance imaging (MRI) is commonly used for the diagnosis of nasopharyngeal carcinoma (NPC) and occipital clivus (OC) invasion, but a proportion of lesions may be missed using non-enhanced MRI. The purpose of this study is to investigate the diagnostic performance of synthetic magnetic resonance imaging (SyMRI) in differentiating NPC from nasopharyngeal hyperplasia (NPH), as well as evaluating OC invasion.

**Methods:**

Fifty-nine patients with NPC and 48 volunteers who underwent SyMRI examination were prospectively enrolled. Eighteen first-order features were extracted from VOIs (primary tumours, benign mucosa, and OC). Statistical comparisons were conducted between groups using the independent-samples t-test and the Mann–Whitney U test to select significant parameters. Multiple diagnostic models were then constructed using multivariate logistic analysis. The diagnostic performance of the models was calculated by receiver operating characteristics (ROC) curve analysis and compared using the DeLong test. Bootstrap and 5-folds cross-validation were applied to avoid overfitting.

**Results:**

The T1, T2 and PD map-derived models had excellent diagnostic performance in the discrimination between NPC and NPH in volunteers, with area under the curves (AUCs) of 0.975, 0.972 and 0.986, respectively. Besides, SyMRI models also showed excellent performance in distinguishing OC invasion from non-invasion (AUC: 0.913–0.997). Notably, the T1 map-derived model showed the highest diagnostic performance with an AUC, sensitivity, specificity, and accuracy of 0.997, 96.9%, 97.9% and 97.5%, respectively. By using 5-folds cross-validation, the bias-corrected AUCs were 0.965–0.984 in discriminating NPC from NPH and 0.889–0.975 in discriminating OC invasion from OC non-invasion.

**Conclusions:**

SyMRI combined with first-order parameters showed excellent performance in differentiating NPC from NPH, as well as discriminating OC invasion from non-invasion.

**Supplementary Information:**

The online version contains supplementary material available at 10.1186/s12880-023-00968-w.

## Background

Nasopharyngeal carcinoma (NPC) is an aggressive head and neck cancer with high incidence rates in several provinces in south-eastern China [1]. Due to the deep location of the nasopharynx and occult symptoms of NPC, early diagnosis is to some extent difficult. With excellent soft-tissue resolution, MRI is the preferred imaging modality for NPC diagnosis, staging, and treatment monitoring. However, NPC and benign nasopharyngeal mucosal overlap to some extent [2–4] and asymmetric hyperplasia causes up to 14% misdiagnosis rate [5], which poses a challenge to the detection of NPC. Moreover, occipital clivus (OC) invasion, which is a poor survival factor [6], occurs in 45.3% NPC patients at the time of diagnosis [7, 8]. Notably, invaded OC typically shows low-intensity on T1WI, but normal OC exhibits age-related changes from uniformly low to uniformly high intensity [9, 10], leading to increased difficulty in discriminating OC invasion. Accordingly, accurate diagnosis of NPC and OC invasion is vital of importance, by developing advanced imaging techniques.

Recently, synthetic magnetic resonance imaging (SyMRI), based on a multiple-delay multiple-echo (MDME) sequence, could generate multiple contrast images in a single scan without contrast agents. Besides, SyMRI could simultaneously provide the longitudinal and transverse relaxation times (T1 and T2) and proton density (PD) of tissues, which are the basic intrinsic properties of MRI physics and independent from the MRI scanners or scanning parameters at a given field strength [11]. Microstructural differences and histopathological information within lesions could be accurately reflected by these three quantitative values [11, 12]. Recently, many studies have demonstrated the value of SyMRI in nasopharynx, breast, bladder and rectal cancer [13–18]. However, it should be noted that most studies used only the Mean value parameter, and additional information such as the tumour heterogeneity, which could be reflected by first-order features, may have been ignored.

First-order features and analysis, which are relatively easier to understand and implement compared with radiomics, reflect the distribution of voxel intensity with high reproducibility [19, 20]. Previous studies [21–24] showed that whole-tumour first-order features, such as Skewness, Variance and so on, can potentially be used for diagnosis and survival prediction in NPC. The research in SyMRI demonstrated that Mean value could help in discriminating NPC from NPH, but the test–retest repeatability and tumour heterogeneity were not evaluated [13]. To avoid the instability in the feature selection step of radiomics [25], we used first-order features as a complement to the Mean value [19]. Therefore, the purposes of our study are to explore whether first-order features derived from SyMRI could effectively discriminate NPC from NPH, and to explore the performance of SyMRI in evaluating OC invasion.

## Methods

### Study participants

The study was approved by the Ethics Committee of our hospital, and written informed consent was obtained from all participants prior to any research activities, including MRI examination. From August 2018 to May 2019, 62 consecutive patients were prospectively enrolled. The inclusion criteria were as follows: (1) nasopharyngoscopy- and biopsy-confirmed NPC; (2) no treatments related to the tumour before the MRI examination; and (3) no concurrent tumours. Three patients were excluded based on the following criteria: (1) the maximum short diameter of the tumour was smaller than 0.5 cm (n = 2); and (2) unqualified images on MRI images (n = 1). Finally, a total of 59 NPC patients were included in this research.

In addition, 49 volunteers were recruited from August to September 2019. The inclusion criteria were as follows: (1) no history of cancer; (2) no symptom related to nasopharyngeal disease; (3) the thickness of nasopharyngeal mucosa more than 3 mm on conventional nonenhanced MRI. Since all the volunteers did not accept the endoscopy, a minimum of 2 years of clinical follow-up was conducted and no suspected tumour in nasopharynx was found. Finally, 48 volunteers were included in this study, and only one was excluded due to unqualified images.

### MRI protocol

All MRI examinations were performed on a 3 T scanner (Discovery MR 750, GE Healthcare, Milwaukee, WI, USA) with an 8-channel head and neck phase array coil. Detailed information on conventional MRI and SyMRI protocol is listed in Table 1. SyMRI scan was performed without contrast. Volunteers did not undergo enhanced scans.

**Table 1:** Imaging parameters for MRI protocol

Parameters	Axial T1WI	Axial T2WI/FS	DWI	Enhanced axial T1WI/FS	SyMRI
Sequence	FSE	FSE	EPI	FSE	MAGiC
TR (ms)	482	6100	2930	250	6200
TE (ms)	13.6	85	80	13.63	18.9/94.7
FOV (cm)	26	26	26	26	26
Acquisition matrix (phase × frequency)	256 × 320	256 × 288	128 × 96	256 × 320	256 × 320
Slice thickness/gap (mm)	4.0/0.4	4.0/0.4	4.0/0.4	4.0/0.4	4.0/0.4
NEX	2.0	2.0	4.0	2.0	1.0
Acquisition time (min)	4.02	4.41	1.22	3.47	7.02

MRI, magnetic resonance imaging; T1WI, T1-weighted Imaging; T2WI, T2-weighted Imaging; FS, fat suppression; DWI = diffusion-weighted imaging; SyMRI, synthetic magnetic resonance imaging; FSE = fast spin echo; EPI = echo planar imaging; NEX = number of excitations; TR = repetition time; TE = echo time; FOV = field of view; MAGiC = magnetic resonance image compilation

### Image processing and segmentation

The acquired raw images were processed using SyMRI software (version 8.0, Synthetic MR, Linkoping, Sweden) to generate three quantitative maps (T1 map, T2 map, and PD map) and multiple contrast-weighted images (T1WI, T2WI, T1WI FLAIR, T2WI FLAIR, short inversion recovery (STIR) and PDWI). The ITK-SNAP software (version 2.2.0, www.itksnap.org, open-source software) was used to segmentation. For each patient, radiologists 1 and 2 (X.Y., Y.L.; 18 and 5 years of tumour imaging experience, respectively) manually delineated volumes of interest (VOIs) along the border of the primary tumour/benign mucosa slice-by-slice, excluding obvious necrosis and cystic areas. Other image contrasts, including conventional T2WI with fat suppression and contrast-enhanced T1WI, were used as references.

The OC invasion was considered positive in cases: low-intensity on T1WI, hyperintensity on T2WI and enhancement on contrast-enhanced T1WI with fat-suppressed, as well as erosion or sclerosis of the bone cortex on CT [26–28]. The diagnosis obtained from MRI imaging was used when there had difference among CT and MR image findings for bone marrow invasion. Radiologists 3 and 4 (M.L., F.Y.; 21 and 3 years of tumour imaging experience, respectively) independently evaluated OC invasion with reference to CT and MRI, and any discordance was resolved by discussion. Then the VOIs of invaded OC in NPC and normal OC in volunteers were delineated on SyT1WI by both radiologists 3 and 4. The data from senior radiologists (X.Y. and M.L.) were used for further analysis.

### First-order features extraction

First-order features were successively extracted from the T1 map, T2 map and PD map of the SyMRI, specifically: the 10th and 90th percentiles, Energy, Entropy, Interquartile Range (IQR), Kurtosis, Maximum, Mean Absolute Deviation (MAD), Mean, Median, Minimum, Range, Robust Mean Absolute Deviation (rMAD), Root Mean Squared (RMS), Skewness, Total Energy, Uniformity, and Variance. In total, 54 first-order features were obtained for each VOI.

### Statistical analysis

All statistical analyses were conducted by IBM SPSS Statistics for Macs (version 26.0, Chicago, IL) and R (version 1.3.1073, R Foundation, Vienna, Austria). A two-tailed *P* < 0.05 indicated statistical significance. Interobserver consistency was analysed using a two-way random interclass correlation (ICC), and first-order features with ICC < 0.8 were excluded.

To compare NPC and NPH, as well as OC invasion and OC non-invasion, we first used either the independent-samples t-test or the Mann–Whitney U test to compare the first-order features according to the normal distribution proved by Kolmogorov–Smirnov test. Then receiver operating characteristic (ROC) curves and Spearman correlation analysis were performed for all significant features. Redundant features were removed if there was a high correlation (r > 0.80) and a relatively lower area under the curve (AUC). Binary logistic regression analysis with backward selection was used to select features and construct models of each imaging contrast. The goodness-of-fit was assessed by the Hosmer–Lemeshow (HL) test. The AUC, sensitivity, specificity and accuracy of models were obtained through ROC analysis. DeLong’s test was used to compare the differential diagnostic performance of the models. To verify the stability of the models, we used two parallel methods through package “rms” and “caret” of R language: (1) Bootstrap resampling method (n = 1000) to plot calibration curves; (2) 200 times 5-folds cross validation to establish the bias-corrected AUCs.

## Results

### Clinical characteristics

The clinical characteristics of the participants are listed in Table 2. Among the patients with NPC, 11, 12, 21, and 15 patients had T1, T2, T3, and T4 stage disease of the primary tumour, respectively, according to the 8th edition of the American Joint Committee on Cancer (AJCC) staging system [29]. Thirty-two (54.2%) patients had OC invasion. All first-order features have excellent consistency (all ICC ≥ 0.802, Additional file 1).

**Table 2:** Clinical characteristics and population demographics of participants

Characteristics	NPC	Volunteers	*P*
Gender			0.271
Male	45 (76.3%)	32 (66.7%)	
Female	14 (23.7%)	16 (33.3%)	
Age	51 (18–68)	43 (28–65)	0.186
Histology		NA	NA
Non-keratinising	21 (35.6%)		
Differentiated			
Non-keratinising	34 (57.6%)		
Undifferentiated			
Keratinizing squamous cell carcinoma	2 (3.4%)		
Unknown	2 (3.4%)		
T stage		NA	NA
T1	11 (18.6%)		
T2	12 (20.3%)		
T3	21 (35.7%)		
T4	15 (25.4%)		
OC invasion		NA	NA
Yes	32 (54.2%)		
No	27 (45.8%)		

Continuous data were expressed as median and range and categorical variables were expressed as percentage

NPC, nasopharyngeal carcinoma; NA, not appliable; OC, occipital clivus

### First-order texture analysis for differentiating NPC tumours from NPH in volunteers

Three unique, diagnostic models each from single, different functional maps and specific first-order features were derived: (1) the PD map (PD_90th percentile and PD_Total Energy); (2) the T1 map (T1_Median, T1_Range and T1_Total Energy); and (3) the T2 map (T2_Minimum and T2_Total Energy). The T2 map-derived model had the best differential value with an AUC of 0.986. There were no significant differences among the three single functional models. The AUC, sensitivity, specificity and accuracy of the PD, T1 and T2 map-derived models are shown in Table 3. The bias-corrected AUCs were 0.965–0.984 (Additional file 1).

**Table 3:** ROC curve analysis of three single functional models in differentiation between NPC and NPH in volunteers

Parameters	NPC	NPH	*P*	Model fit	AUC (95% CI)	Sensitivity%	Specificity%	Accuracy%
*T1 map derived model*				0.870	0.972 (0.948, 0.996)	88.1	95.8	88.8
T1_Median	1405.10 (1366.50, 1519.00)	1617.13 (1464.85, 1761.93)	0.000					
T1_Range	2718.00 (2170.00, 3256.40)	2357.80 (1910.30, 3016.78)	0.041					
T1_Total Energy (× 10^6^)	8800.08 (5866.79, 13,639.98)	1682.82 (1083.85, 2798.88)	0.000					
*T2 map derived model*				1.000	0.986 (0.970, 1.000)	96.6	93.7	94.4
T2_Minimum	53.70 (47.10, 59.20)	65.20 (62.93, 68.08)	0.000					
T2_Total Energy (× 10^6^)	27.36 (20.71, 40.30)	5.18 (2.88, 7.80)	0.000					
*PD map derived model*				0.890	0.975 (0.952, 0.998)	89.8	95.8	90.7
PD_90th percentile	100.10 (97.7, 100.7)	101.59 (100.86, 102.12)	0.000					
PD_Total Energy (× 10^6^)	29.01 (21.29, 49.53)	5.12 (3.16, 7.75)	0.000					

Data was expressed as median (interquartile range)

ROC, receiver operating characteristic; NPC, nasopharyngeal carcinoma; AUC, area under the curve; 95% CI, 95% confidence interval

### First-order texture analysis for differentiation between OC invasion and OC non-invasion

The OC invasion group (n = 32) had higher T1_Mean, T1_Total Energy and T2_MAD, lower PD_Maximum, T2_10th percentile and T2_Energy than the OC non-invasion group of volunteers (n = 48). The T1 map-derived model showed higher diagnostic performance than the T2 map-derived model (*P* = 0.007), and there were no significant differences in AUC between the comparisons of the other single map models (Table 4). The bias-corrected AUCs were 0.889–0.975 (Additional file 1).

**Table 4:** ROC curve analysis of three single functional models in discrimination between OC invasion and OC non-invasion

Models	OC invasion	OC non-invasion	*P*	Model fit	AUC (95% CI)	Sensitivity%	Specificity%	Accuracy%
*T1 map derived model*				1.000	0.997 (0.990, 1.000)	96.9	97.9	97.5
T1_Mean	1570.61 (1348.53, 1892.24)	651.15 (588.75, 756.02)	0.000					
T1_Total Energy (× 10^6^)	3621.43 (1488.39, 7822.86)	1398.09 (1048.77, 2008.46)	0.000					
*T2 map derived model*				0.885	0.913 (0.851, 0.975)	93.8	72.9	80.0
T2_10th percentile	72.85 (61.46, 80.28)	83.06 (75.80, 89.22)	0.000					
T2_Energy (× 10^6^)	12.14 (7.51, 32.71)	33.98 (18.90, 54.38)	0.000					
T2_MAD	15.96 (11.83, 20.44)	12.79 (10.95, 14.03)	0.002					
*PD map derived model*				0.209	0.975 (0.945, 1.000)	93.7	95.8	93.8
PD_Maximum	107.00 (105.00, 108.15)	133.5 (122.95, 140.05)	0.000					

Data was expressed as median (interquartile range)

ROC, receiver operating characteristic; OC, occipital clivus; AUC, area under the curve; 95% CI, 95% confidence interval; MAD, mean absolute deviation

**Fig. 1:**
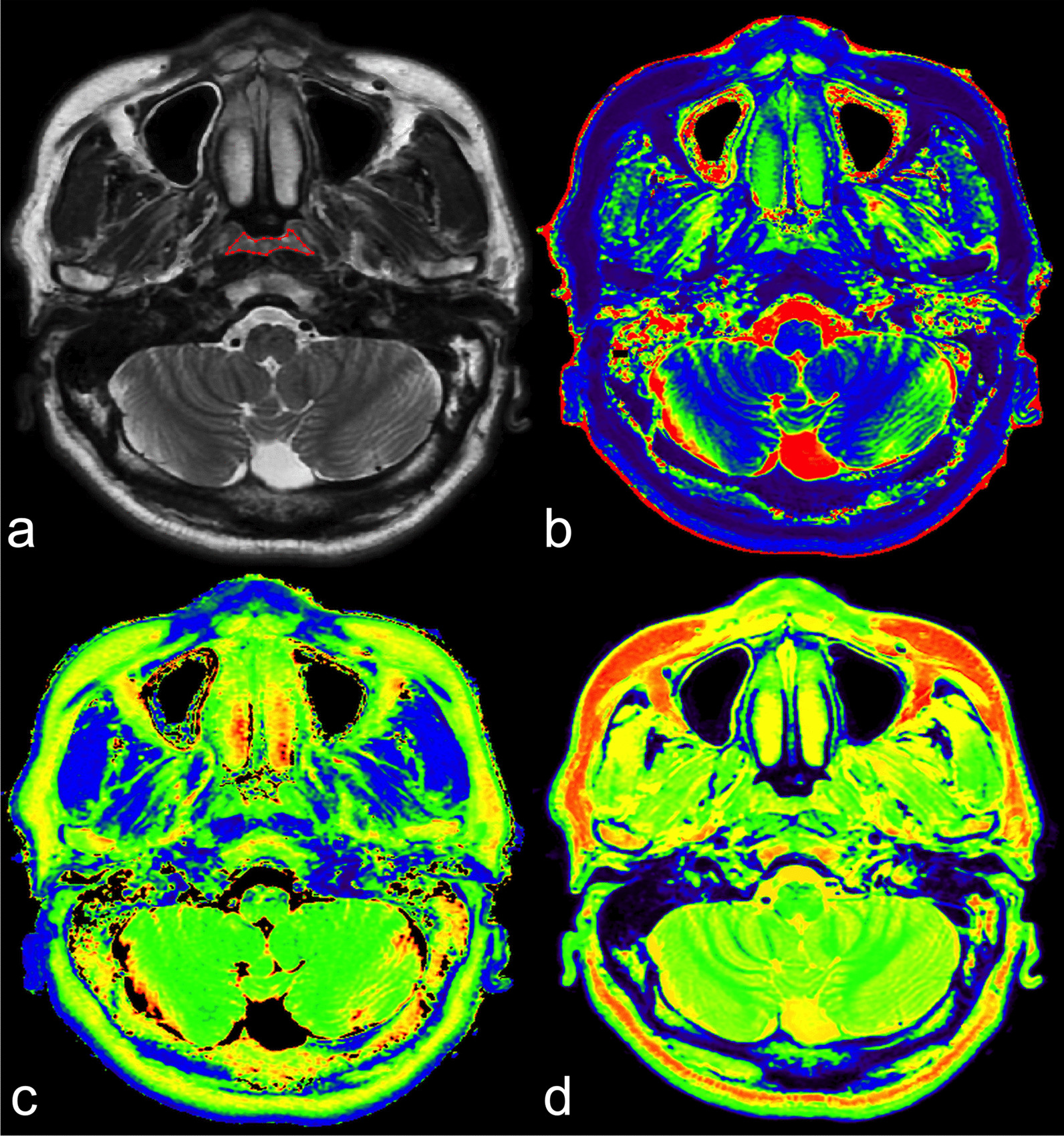
SyMRI of a 48-year-old male NPC patient. **a** Axial T2WI shows uniform thickening with heterogeneous iso-slightly high signal in the posterior wall of the nasopharyngeal cavity, and bilateral pharyngeal recess are collapsed. VOI was manually delineated along the border of the tumour slice by slice. T1 map (**b**), T2 map (**c**) and PD map (**d**) at the same level as in (**a**). SyMRI, synthetic magnetic resonance imaging; NPC, nasopharyngeal carcinoma; T2WI, T2-weighted image; VOI, volume of interest; PD, proton density

**Fig. 2:**
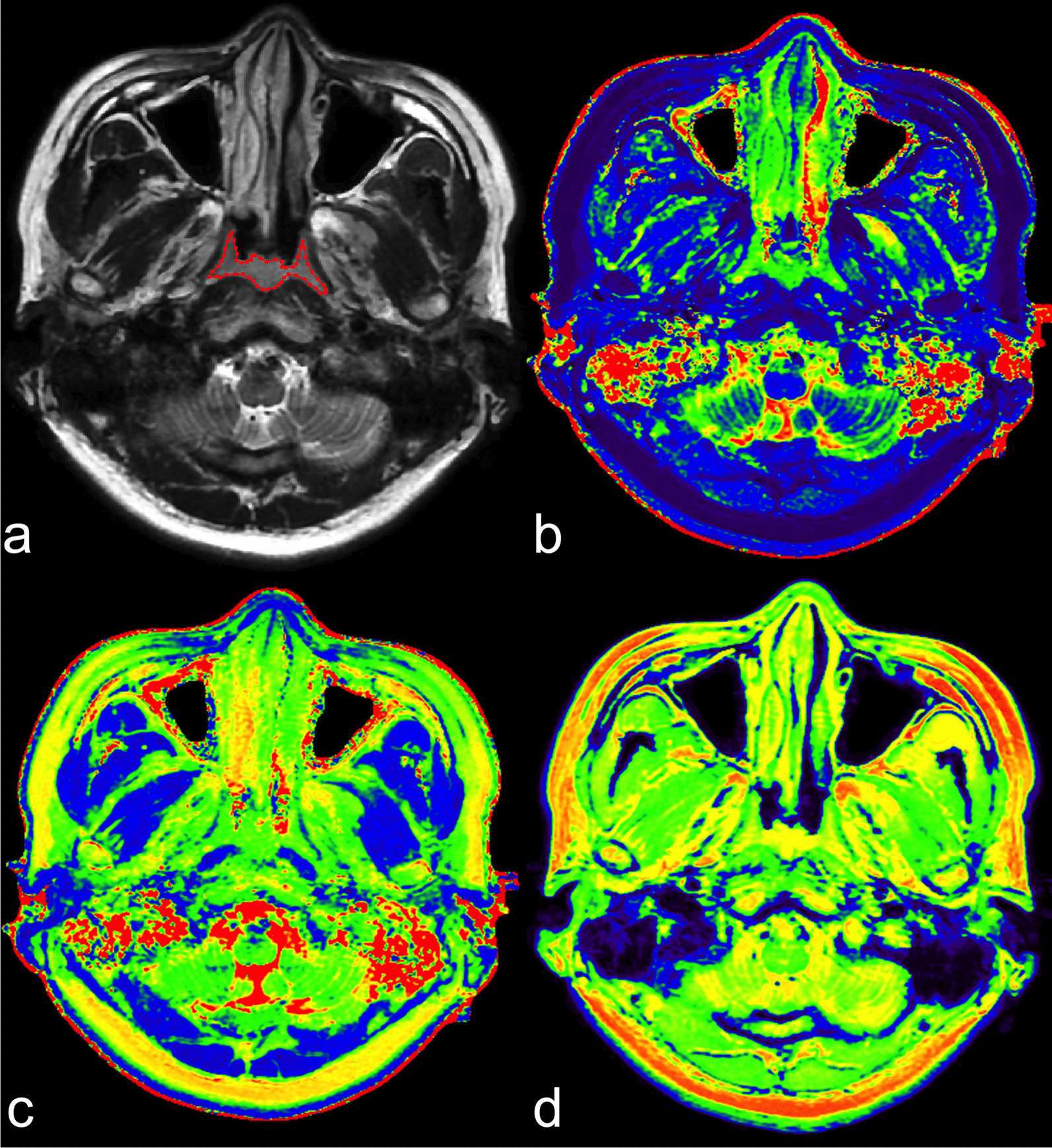
SyMRI of a 38-year-old female volunteer. **a** Axial T2WI shows hyperenhancing tissue along the posterior nasopharyngeal wall and bilateral lateral nasopharyngeal wall. Bilateral pharyngeal recess are collapsed and show symmetric wall thickness. VOI was manually delineated along the border of the benign mucosa slice by slice. Derived T1 map (**b**), T2 map (**c**) and PD map (**d**). SyMRI, synthetic magnetic resonance imaging; T2WI, T2-weighted image; VOI, volume of interest; PD, proton density

**Fig. 3:**
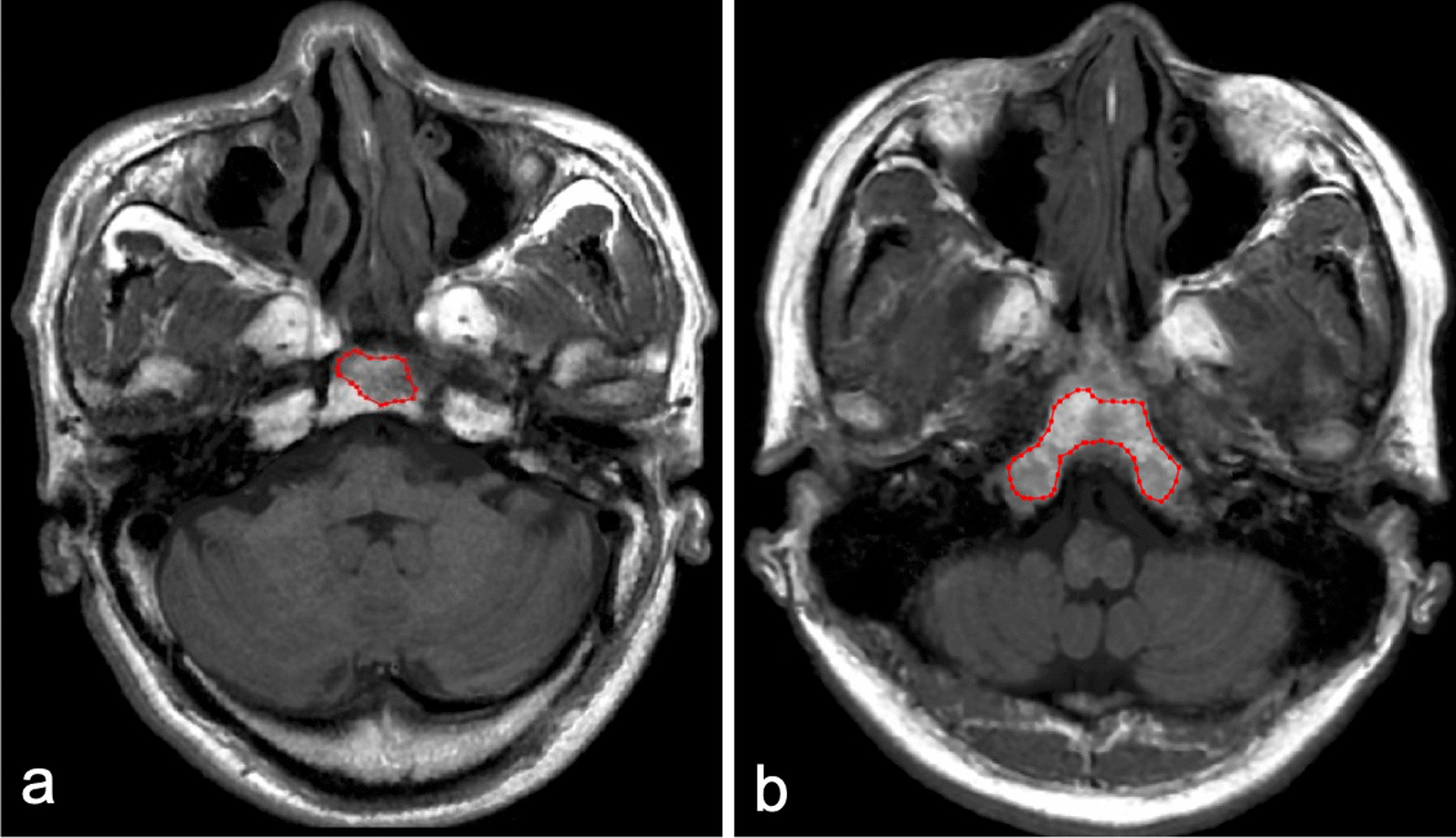
Representative images of OC invasion and OC non-invasion. Invaded OC in 56-year-old NPC patient (**a**) shows low signal on axial T1WI of SyMRI, which shows similar signal characteristics with non-invaded OC in 38-year-old volunteer (**b**). VOI was manually delineated along the border of invaded OC and non-invaded OC on axial T1WI of SyMRI. OC, occipital clivus; NPC, nasopharyngeal carcinoma; T1WI, T-weighted image; SyMRI, synthetic magnetic resonance imaging; VOI, volume of interest

**Fig. 4:**
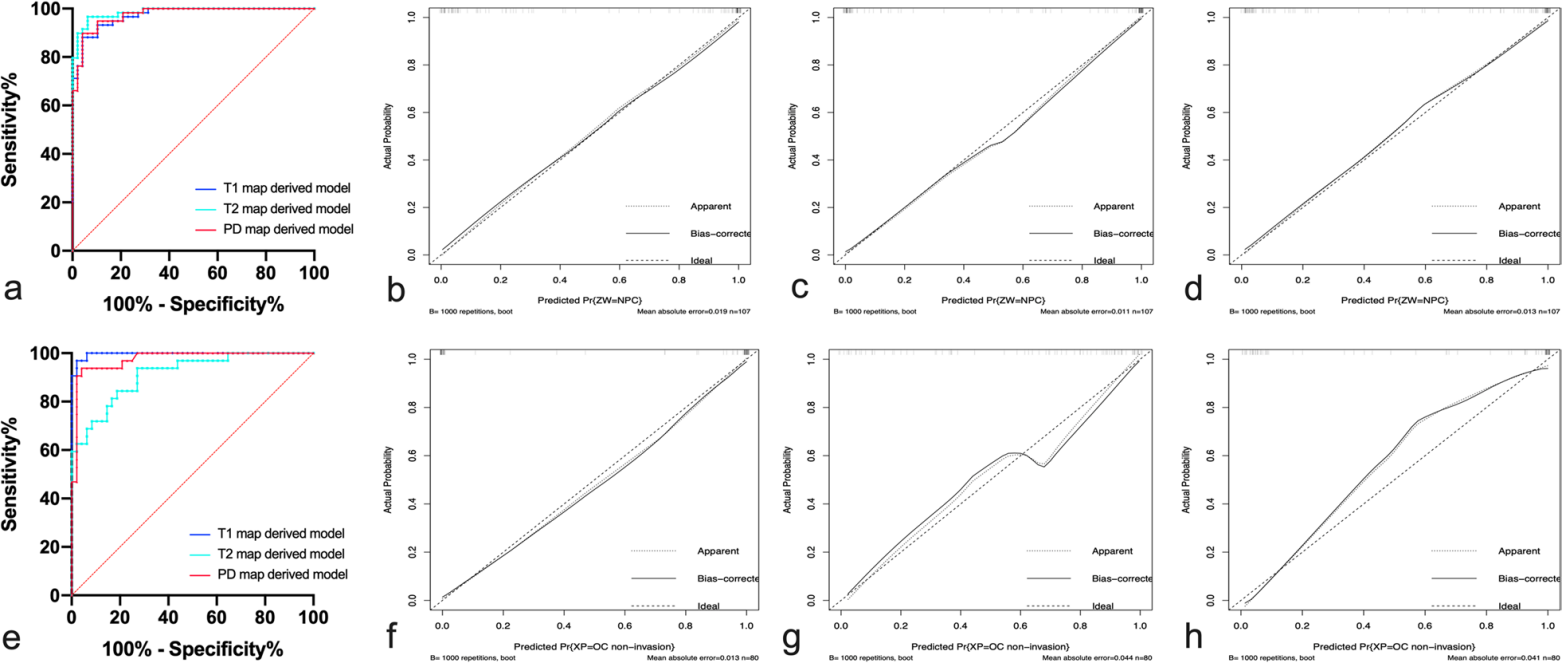
ROC curves (**a, e**) and calibration curves (**b-d**, **f–h**) of models in all sample. **a** ROC curves of diagnostic models based on T1, T2 and PD maps for differentiating NPC from NPH in volunteers. Calibration curves of nomogram developed in T1 map (**b**), T2 map (**c**) and PD map (**d**) derived model. **e** ROC curves of diagnostic models based on T1, T2 and PD maps for the differentiation between OC invasion and OC non-invasion. Calibration curves of nomogram developed in T1 map (**f**), T2 map (**g**) and PD map (**h**) derived model. ROC, receiver operating characteristic; PD, proton density; NPC, nasopharyngeal carcinoma; OC, occipital clivus

Typical cases of NPC and volunteer are illustrated in Figs. 1 and 2. Representative images of OC invasion and OC non-invasion are illustrated in Fig. 3. The comparisons between these three single functional models and calibration curves are presented in Fig. 4.

## Discussion

To our knowledge, this is the first prospective study to investigate the role of SyMRI combined with first-order features for the diagnosis of NPC. Our results demonstrated that the T1, T2 and PD relaxation times of NPC derived from SyMRI images were significantly different from those for NPH. Moreover, in OC invasion discrimination, the T1 map-derived model showed the highest diagnostic performance. These findings indicate that SyMRI could reflect the changes in the intrinsic characteristics of benign nasopharyngeal mucosa progressing to a tumour. These quantitative parameters provide auxiliary value for the morphology, which makes the evaluation more objective.

Endoscopic biopsy is the gold standard for diagnosis of NPC. However, this examination method is invasive and it may miss some lesions generally attributed to submucosal location, coexistent hyperplasia, and difficult pharyngeal recess structure [30, 31]. About 12% of NPC invisible under endoscopy can be successfully detected by MRI [32]. Conventional morphology on MRI, such as location site, symmetry, stripes and signal intensity could be valuable in differentiating NPC from NPH [30]. However, these image features are susceptible to the subjective influence of observers, and contrast agents are required. Recently, radiomics based on T2WI/FS provide a quantitative method to diagnose NPC, but instability in the feature selection step could reduce its reliability [25]. The quantitative parameters of various functional MRIs have also been applied to identify NPC from NPH, and higher K_trans_ and blood flow, and lower diffusion parameters (including ADC, D, f) were found in NPC [30, 33, 34]. Moreover, Meng et al. [13] found Mean of T1, T2 and PD maps derived from SyMRI could help in discriminating NPC from NPH, but the performance of PD_Mean was poor (AUC = 0.624) and percentile parameter has an advantage than Mean parameter [35, 36]. Conducted in a non-endemic area, our study increased the sample size and included tumour heterogeneity to further explore the performance of SyMRI in diagnosing NPC, as well as OC invasion.

T1, T2, and PD values reflect the intrinsic properties of tissues. The higher the cellularity is, the lower the extracellular space and free water content, thereby resulting in a reduction in the T1 and T2 values [37]. The 90th percentile, Median and Minimum represent the grey-level intensity within the VOIs. Our results found that NPC had significantly lower T1_Median and T2_Minimum values than NPH. A possible reason was the abundance of mucus in the benign nasopharyngeal mucosa, which correlates with higher T1 and T2 values. Similar results were found in previous studies on NPC vs. NPH, as well as prostate cancer vs. peripheral zone/benign prostatic hyperplasia and cervical cancer vs. normal mucosa [11, 13, 38]. However, recent study [15] found that the mean T1 value of breast malignancy was significantly higher than that of benign tumours, possibly because the ROI of benign tumours included more fat tissue. Thyroid research also showed that papillary thyroid carcinoma had a higher T2 value than contralateral normal tissue [39]. These controversial results stated that the different control groups led to different variations.

The PD value represents the apparent concentration of water protons (mobile hydrogen atoms) in each voxel [40]. When malignant cells invade normal tissue, the density of lesions increases, and the water content decreases, resulting in reduced PD values. The lower PD value also correlates with worse biological tumour behaviour [11, 16]. A study in breast cancer [41] found lower PD values in malignancies than in benign tumours. Our study obtained consistent results, given that several PD values (the 10th, 90th percentile, Mean, Median, Minimum) in NPC were lower than those in NPH, with the most representative being the PD_90th percentile. Due to statistically 90th percentile is less influenced by random fluctuations than Mean value [35], parameter 90th percentile has an advantage over the Mean, which may be one of reasons for the poor performance of PD_Mean in previous study [13].

OC invasion at initial diagnosis is a poor factor for survival of T3 stage NPC patients [6]. Previously studies found the signal intensity of OC is related to age and gender [9], and abnormal residual OC signal could persist several years after radiotherapy without recurrence [27], which make the identification of OC invasion and recurrence somewhat difficult. In addition, according to the criteria reported in studies [26–28], contrast-enhanced sequences are an indispensable examination, so false-positive is more likely to occur in people who only performed non-enhanced scans. Our study demonstrated that SyMRI without contrast agents could objectively evaluate OC invasion. Compared to the OC non-invasion group, the OC invasion group had a higher T1_Mean and lower PD_Maximum and T2_10th percentile, while T1_Mean was the most representative parameter. Cancer cells infiltrate the trabecular architecture and replace the fatty and haematopoietic marrow in normal bone, resulting in increased T1 values and decreased PD and T2 values, which is supported by studies of bone metastasis [42–44]. The MAD parameter may represent tumour heterogeneity, and a higher T2_MAD in the OC invasion group reflects a more heterogeneous tumour structure than that of the OC non-invasion group.

Energy and Total Energy, as larger values, imply a greater sum of the squares of these values and volume-confounded, are thus positively correlated with the magnitude of voxel value and the volume of delineation. In our study, NPC had lower T1, T2 and PD values but higher Total Energy, while OC invasion had a higher T1 value and higher T1_Total Energy, but lower T2 value and lower T2_Energy. We speculated that the volume may greatly impact this formal comparison, since there is a large difference in the parameter volume between NPC tumours and NPH (5945.12 mm^3^ vs. 1403.05 mm^3^) but relatively small between OC invasion and non-invasion group (1674.28 mm^3^ vs. 3281.64 mm^3^). Zhang et al. [45] found stage IA endometrial carcinoma had lower signal intensities (including Mean, Median and so on) and Total Energy on ADC map than benign endometrial lesions. Ghosh et al. [46] proved that neuroblastomas with MYCN amplification had higher signal intensities (Mean and Maximum) and Energy on ADC map than tumours without MYCN amplification. Zhao et al. [47] also revealed that rectal cancer with lymph node metastasis had both a higher signal intensity (Maximum of the T2 and PD maps) and Energy than cancer without lymph node metastasis. The mapping of values in those three studies was consistent with Energy/Total Energy, so the effect of volume is not mentioned. However, according to our results, we believe that both the mapping value and the volume of the object must be considered when evaluating Energy and Total Energy.

There were several limitations in this study. First, the study sample was relatively small and was not divided into training and validation groups before the model was constructed. We recruited volunteers as control group, who did not perform nasopharyngoscopy and may have nasopharyngeal hyperplasia or inflammation. Therefore, a minimum of 2 years follow-up was conducted on control group, and no tumour sign was found. Due to the spectral deviation of diseases in our hospital and the incidence of tumours, other malignancies (such as lymphoma and adenoid cystic carcinoma) were not included in our research. Second, this was a single-centre study using a certain type of MRI scanner with an identical protocol. Multicentre studies with different MRI scanners and protocols are warranted before our findings are introduced into routine clinical practice. Last, due to the limited number of samples, the tumour stage, histological types and grades of NPC could not be considered, and a comparison between early-stage NPC and NPH could not be conducted in this study. A larger sample size is needed to investigate the correlation between the quantitative parameters of SyMRI and those factors.

## Conclusions

The first-order parameters from SyMRI were significantly different between NPC and NPH and between OC invasion and non-invasion. Consequently, SyMRI was shown to be a reliable and useful tool, not only to provide additional quantitative value based on morphology for the diagnosis of NPC but also for evaluating the development of tumours by quantitatively measuring the changes in intrinsic characteristics in tissues.

## Supplementary Information

**Additional file 1: Supplementary Table 1.** Comparison of SyMRI histogram parameters between NPC and NPH. **Supplementary Table 2.** Comparison of SyMRI histogram parameters between OC invasion group and OC non-invasion group. **Supplementary Table 3.** AUC analysis and 5-folds Cross-Validation for models.

## Data Availability

The datasets used and analysed during the current study are available from the corresponding author on reasonable request.

## Abbreviations

NPC: Nasopharyngeal carcinoma
ROC: Receiver operating characteristic
AUC: Area under the curve
OC: Occipital clivus
SyMRI: Synthetic magnetic resonance imaging
PD: Proton density
RMS: Root mean squared
VOI: Volumes of interest
ICC: Interclass correlation
