# Supplementary material for: The utility of texture analysis based on quantitative synthetic magnetic resonance imaging in nasopharyngeal carcinoma: a preliminary study
Source: BMC Med Imaging. 2023 Jan 25;23:15. doi: 10.1186/s12880-023-00968-w (PMC9875491; doi:10.1186/s12880-023-00968-w)
Supplement: Supplementary file 1 — Additional file 1: Supplementary Table 1. Comparison of SyMRI histogram parameters between NPC and NPH. Supplementary Table 2. Comparison of SyMRI histogram parameters between OC invasion group and OC non-invasion group. Supplementary Table 3. AUC analysis and 5-folds Cross-Validation for models. [file 12880_2023_968_MOESM1_ESM.docx]

**Supplementary Table 1 Comparison of SyMRI histogram parameters between NPC and NPH**

| First-order parameters | T1 map | | | | T2 map | | | | PD map | | | |
| --- | --- | --- | --- | --- | --- | --- | --- | --- | --- | --- | --- | --- |
|  | NPC | NPH | P | ICC^#^ | NPC | NPH | P | ICC^#^ | NPC | NPH | P | ICC^#^ |
| 10th Percentile | 1184.050  (1120.920-1244.000) | 1267.800  (1130.855-1351.158) | 0.152* | 0.912 | 71.520  (66.300-75.700) | 77.415  (73.810-80.100) | 0.000* | 0.978 | 79.200  (76.200-81.700) | 82.900  (77.850-86.420) | 0.001* | 0.948 |
| 90th Percentile | 1865.600  (1627.780-2044.260) | 2110.790  (1982.315-2367.610) | 0.000* | 0.981 | 99.500  (93.900-107.200) | 102.760  (97.310-106.575) | 0.139 | 0.981 | 100.100  (97.700-100.700) | 101.590  (100.855-102.115) | 0.000 | 0.989 |
| Energy (×  10^6^) | 9616.631  (6066.979-1405.284) | 1740.246  (1120.830-  2894.385) | 0.000 | 0.992 | 31.065  (20.325-46.714) | 5.203  (2.978-8.064) | 0.000 | 0.994 | 33.820  (21.135-53.481) | 5.298  (3.271-7.855) | 0.000 | 0.994 |
| Entropy | 5.025  (4.666-5.326) | 5.345  (5.174-5.631) | 0.000* | 0.945 | 0.903  (0.679-1.081) | 0.863  (0.559-1.042) | 0.358* | 0.978 | 0.892  (0.685-0.952) | 0.978  (0.870-1.022) | 0.001 | 0.961 |
| Interquartile Range | 305.350  (232.750-397.950) | 480.100  (403.556-537.625) | 0.000 | 0.945 | 14.400  (12.900-16.900) | 12.738  (10.300-15.525) | 0.014* | 0.936 | 10.600  (9.600-12.725) | 11.413  (7.725-13.000) | 0.442* | 0.947 |
| Kurtosis | 7.033  (5.015-9.549) | 3.793  (2.952-5.684) | 0.000 | 0.802 | 8.921  (4.141-16.775) | 4.230  (3.038-7.279) | 0.000 | 0.983 | 3.417  (2.862-4.265) | 3.646  (2.874-6.750) | 0.172 | 0.891 |
| Maximum | 3509.600  (2999.000-3997.300) | 3240.550  (2823.550-3816.875) | 0.089* | 0.877 | 181.900  (156.000-239.200) | 134.450  (120.050-171.650) | 0.000 | 0.950 | 106.300  (105.200-108.000) | 105.600  (104.025-107.325) | 0.106 | 0.813 |
| MAD | 214.730  (160.368-269.404) | 293.026  (256.336-346.287) | 0.000 | 0.944 | 9.340  (8.364-10.838) | 8.207  (6.610-10.352) | 0.010 | 0.930 | 6.256  (5.619-6.928) | 6.440  (5.458-7.415) | 0.911* | 0.906 |
| Mean | 1475.946  (1397.848-1596.578) | 1680.575  (1499.589-1836.773) | 0.000 | 0.982 | 85.106  (79.640-90.170) | 90.095  (86.216-93.621) | 0.001* | 0.989 | 90.135  (86.824-91.752) | 94.258  (90.566-96.147) | 0.000* | 0.980 |
| Median | 1405.100  (1366.500-1519.000) | 1617.125  (1464.850-1761.925) | 0.000 | 0.985 | 83.200  (79.100-88.300) | 88.100  (85.225-90.800) | 0.000* | 0.990 | 90.500  (87.400-92.500) | 96.400  (92.625-99.975) | 0.000 | 0.983 |
| Minimum | 800.500  (672.000-907.700) | 829.100  (729.800-968.500) | 0.088* | 0.852 | 53.700  (47.100-59.200) | 65.200  (62.925-68.075) | 0.000* | 0.954 | 51.600  (44.900-62.500) | 62.450  (53.225-67.450) | 0.000 | 0.827 |
| Range | 2718.000  (2170.000-3256.400) | 2357.800  (1910.300-3016.775) | 0.039* | 0.842 | 130.300  (106.400-189.600) | 69.400  (54.650-106.425) | 0.000 | 0.951 | 54.000  (45.200-63.500) | 42.850  (37.225-53.175) | 0.000 | 0.819 |
| RMS | 1498.446  (1423.961-1629.447) | 1716.408  (1580.362-1906.119) | 0.000* | 0.983 | 85.904  (81.435-91.432) | 90.799  (86.478-93.621) | 0.003 | 0.976 | 90.421  (87.204-92.077) | 94.603  (91.105-96.415) | 0.000* | 0.981 |
| Skewness | 1.430  (1.094-1.830) | 0.661  (0.293-1.128) | 0.000 | 0.824 | 1.235  (0.679-2.155) | 0.833  (0.314-1.456) | 0.015 | 0.980 | -0.587  (-0.866-  -0.336) | -1.055  (-1.860--0.673) | 0.000 | 0.951 |
| Total Energy (×  10^6^) | 8800.082  (5866.786-1363.998) | 1682.818  (1083.846-2798.879) | 0.000 | 0.992 | 27.363  (20.718-40.303) | 5.179  (2.880-7.797) | 0.000 | 0.994 | 29.008  (21.286-49.530) | 5.123  (3.163-7.746) | 0.000 | 0.994 |
| Uniformity | 0.039  (0.032-0.052) | 0.029  (0.025-0.033) | 0.000 | 0.960 | 0.639  (0.559-0.754) | 0.638  (0.547-0.779) | 0.934* | 0.981 | 0.594  (0.539-0.734 | 0.524  (0.509-0.631) | 0.000 | 0.970 |
| Variance | 92595.252  (51660.113-133786.921) | 136456.620  (108716.534-218018.398) | 0.000 | 0.929 | 153.852  (126.106-273.229) | 111.103  (74.648-176.513) | 0.002 | 0.882 | 60.632  (48.567-77.980) | 61.386  (47.199-82.052) | 0.652 | 0.899 |
| rMAD | 129.515  (99.813-171.138) | 200.175  (170.174-222.400) | 0.000 | 0.948 | 6.102  (5.349-7.080) | 5.368  (4.452-6.473) | 0.016 | 0.943 | 4.476  (4.058-5.088) | 4.849  (3.562-5.434) | 0.684 | 0.941 |

Data was expressed as median (interquartile range).

* indicates independent sample t test; ^#^ indicates the comparison between two radiologists.

NPC, nasopharyngeal carcinoma; PD, proton density; MAD, mean absolute deviation; SyMRI, synthetic magnetic resonance imaging.

**Supplementary Table 2 Comparison of SyMRI histogram parameters between OC invasion group and OC non-invasion group**

| First-order parameters | T1 map |  |  | T2 map |  |  | PD map |  |  |
| --- | --- | --- | --- | --- | --- | --- | --- | --- | --- |
|  | P | AUC (95% CI) | ICC | P | AUC (95% CI) | ICC | P | AUC (95% CI) | ICC |
| 10th Percentile | 0.000 | 0.987 (0.970-1.000) | 0.992 | 0.000* | 0.736 (0.691-0.853) | 0.991 | 0.000* | 0.793 (0.695-0.891) | 0.980 |
| 90th Percentile | 0.000* | 0.990 (0.974-1.000) | 0.997 | 0.912* | 0.541 (0.405, 0.668) | 0.980 | 0.000 | 0.767 (0.659-0.874) | 0.998 |
| Energy | 0.000 | 0.765 (0.636-0.894) | 0.995 | 0.000 | 0.747 (0.632, 0.863) | 0.968 | 0.000 | 0.848 (0.761-0.935) | 0.997 |
| Entropy | 0.000* | 0.976 (0.945-1.000) | 0.998 | 0.002 | 0.702 (0.574-0.830) | 0.974 | 0.000* | 0.768 (0.661-0.874) | 0.947 |
| Interquartile Range | 0.000 | 0.978 (0.953-1.000) | 0.990 | 0.022* | 0.634 (0.501-0.767) | 0.961 | 0.003* | 0.678 (0.550-0.805) | 0.969 |
| Kurtosis | 0.038 | 0.637 (0.507-0.767) | 0.824 | 0.020 | 0.654 (0.527-0.781) | 0.833 | 0.191 | 0.587 (0.445-0.728) | 0.907 |
| Maximum | 0.000 | 0.954 (0.901-1.000) | 0.975 | 0.074* | 0.618 (0.485-0.752) | 0.804 | 0.000 | 0.975 (0.945-1.000) | 0.975 |
| MAD | 0.000 | 0.979 (0.951-1.000) | 0.995 | 0.002 | 0.706 (0.579-0.832) | 0.914 | 0.074* | 0.585 (0.445-0.725) | 0.953 |
| Mean | 0.000 | 0.990 (0.976-1.000) | 0.996 | 0.236* | 0.613 (0.478-0.747) | 0.990 | 0.000* | 0.824 (0.734-0.913) | 0.991 |
| Median | 0.000* | 0.990 (0.976-1.000) | 0.996 | 0.041* | 0.656 (0.527-0.785) | 0.996 | 0.000 | 0.799 (0.703-0.895) | 0.986 |
| Minimum | 0.000 | 0.945 (0.894-0.997) | 0.914 | 0.004* | 0.690 (0.563-0.817) | 0.811 | 0.001* | 0.738 (0.620-0.857) | 0.865 |
| Range | 0.000 | 0.937 (0.872-1.000) | 0.968 | 0.024 | 0.650 (0.521-0.779) | 0.848 | 0.000 | 0.771 (0.668-0.874) | 0.879 |
| RMS | 0.000* | 0.990 (0.974-1.000) | 0.997 | 0.455 | 0.549 (0.406-0.692) | 0.887 | 0.000* | 0.822 (0.731-0.913) | 0.992 |
| Skewness | 0.000 | 0.568 (0.436-0.669) | 0.847 | 0.005 | 0.688 (0.567-0.808) | 0.843 | 0.026 | 0.647 (0.520-0.774) | 0.951 |
| Total Energy | 0.000* | 0.766 (0.638-0.893) | 0.995 | 0.000 | 0.743 (0.625-0.861) | 0.965 | 0.000 | 0.841 (0.753-0.929) | 0.997 |
| Uniformity | 0.000* | 0.975 (0.944-1.000) | 0.997 | 0.031 | 0.643 (0.510-0.776) | 0.983 | 0.000 | 0.801 (0.702-0.901) | 0.956 |
| Variance | 0.000 | 0.980 (0.950-1.000) | 0.993 | 0.000 | 0.736 (0.614-0.857) | 0.817 | 0.498 | 0.544 (0.403-0.687) | 0.948 |
| rMAD | 0.000 | 0.976 (0.946-1.000) | 0.993 | 0.027 | 0.646 (0.514-0.779) | 0.966 | 0.027 | 0.646 (0.514-0.779) | 0.968 |

* indicates independent sample t test.

OC, occipital clivus; PD, proton density; MAD, mean absolute deviation; SyMRI, synthetic magnetic resonance imaging.

**Supplementary Table 3 AUC analysis and 5-folds Cross-Validation for models**

| Models | AUC (95% CI) | Bias-corrected AUC* |
| --- | --- | --- |
| **NPC vs. NPH** | | |
| T1 map derived model | 0.972 (0.948, 0.996) | 0.965 ± 0.033 |
| T2 map derived model | 0.986 (0.970, 1.000) | 0.984 ± 0.019 |
| PD map derived model | 0.975 (0.952, 0.998) | 0.972 ± 0.028 |
| **OC invasion vs. OC non-invasion** | | |
| T1 map derived model | 0.997 (0.990 to 1.000) | 0.961 ± 0.070 |
| T2 map derived model | 0.913 (0.851 to 0.975) | 0.889 ± 0.084 |
| PD map derived model | 0.975 (0.945 to 1.000) | 0.975 ± 0.037 |

*: 200 times 5-folds cross-validation, and the results were expressed as mean and standard deviation.
